# From Ganglion Cell to Photoreceptor Layer: Timeline of Deterioration in a Rat Ischemia/Reperfusion Model

**DOI:** 10.3389/fncel.2019.00174

**Published:** 2019-05-10

**Authors:** Marina Palmhof, Viktoria Frank, Pascal Rappard, Emely Kortenhorn, Julia Demuth, Nora Biert, Gesa Stute, H. Burkhard Dick, Stephanie C. Joachim

**Affiliations:** Experimental Eye Research, University Eye Hospital, Ruhr-University Bochum, Bochum, Germany

**Keywords:** ischemia/reperfusion, ERG, retinal thickness, retinal ganglion cell, timeline, photoreceptor, amacrine cell, bipolar cell

## Abstract

Neuronal damage and impaired vision in different retinal disorders are induced, among other factors, by ischemia/reperfusion (I/R). Since the mechanisms and the progression of ischemic injury are still not completely clarified, a timeline of this retinal degeneration is needed. In this study, we investigated protein and mRNA alterations at 2, 6, 12, and 24 h as well as 3 and 7 days after ischemia to determine the course of an ischemic insult through the whole retina. Moreover, functional analyses were performed at later stages. We detected a significant functional loss of cells in the inner nuclear layer and photoreceptors at 3 and 7 days. Additionally, the thickness of the whole retina was decreased at these points in time, indicating a severe degradation of all retinal layers. Immunohistological and qRT-PCR analyses of retinal ganglion cells (RGCs), glial cells, AII amacrine, cone and rod bipolar as well as cone and rod photoreceptor cells confirmed this first assumption. Our results show that all investigated cell types were damaged by ischemia induction. Especially RGCs, cone bipolar cells, and photoreceptor cones are very sensitive to I/R. These cells were lost shortly after ischemia induction with a progressive course up to 7 days. In addition, Müller cell gliosis was observed over the entire period of time. These results provide evidence, that I/R induces damage of the whole retina at early stages and increases over time. In conclusion, our study could demonstrate the intense impact of an ischemic injury. The ischemic defect spreads across the whole retina right up to the outer layers in the long-term and thus seems to impair the visual perception already during the stimulus processing. In addition, our findings indicate that the cone pathway seems to be particularly affected by this damage.

## Introduction

Retinal neurodegenerative diseases, including AMD, diabetic retinopathy, glaucoma, and retinal vein occlusion, are multi factorial and so far insufficiently investigated. One common pathomechanism of these disorders, which can lead to the visual disturbance through to loss of vision, is ischemia (Kim et al., 2013; Nakahara et al., 2015).

Retinal ischemia is defined as a lack of retinal blood flow. In order to investigate this process experimentally a transient ischemia is induced in animals for a defined period of time with a subsequent natural reperfusion. Different animal models exist to induce transient ischemia and to investigate its impact on the retina. One of the most used models is the so-called pressure-induced I/R model. This model imitates the ischemic events associated with high IOP, as it occurs in glaucoma (Buchi et al., 1991; Weinreb and Khaw, 2004; Vidal-Sanz et al., 2012). The circulatory disorder of the retina is induced in this model by a temporary raise of the IOP, which results in a compression of the blood vessels and thus in a reduced blood supply. The consequence for the several retinal cell types is a lower oxygenation capacity and supply of nutrients followed by the formation of oxidative stress during the recurring blood flow (Kaur et al., 2008; Minhas et al., 2012; Kim et al., 2013). These conditions result in a loss of retinal functionality, inflammation, and retinal tissue damage, including neuronal cell death (Szabo et al., 1991; Dijk et al., 2004a; Kaur et al., 2008; Belforte et al., 2011; Joachim et al., 2012; Minhas et al., 2012; Andreeva et al., 2014; Schmid et al., 2014; Nakahara et al., 2015). Cell death is triggered by different cell death mechanisms such as apoptosis and autophagy (Buchi, 1992; Singh et al., 2001; Rosenbaum et al., 2010; Piras et al., 2011). Several research groups were able to show a functional disorder of the inner retinal cell layers via ERG measurements (Schmid et al., 2014). Furthermore, it is known that RGCs and other inner retinal cells, like amacrine cells, are mainly affected by I/R (Lam et al., 1999; Goldblum and Mittag, 2002; Zheng et al., 2004; Bek, 2009; Nakano et al., 2011; Schmid et al., 2014).

However, most studies only analyzed single points in time after I/R with a focus on the inner retinal layers. There are only a few studies on photoreceptors with distinct results concerning the ischemic impact. For example, Zhao et al. (2013) could not detect any effect on photoreceptors after a 17 min transient global ischemia. They examined the tissue 6, 12, and 48 h after ischemia, but neither immunoreactivity nor fluorescent density of rod and cone markers were altered due to ischemia. Apart from the fact that the degree and duration of an ischemia induction also affect the severity of the damage, it is assumed that photoreceptors are more tolerant against ischemia. However, it is necessary to draw up a temporal progress of degeneration including the investigation of the outer retinal layers for a better understanding, as the mechanism of ischemic neuronal damage is not yet fully researched and understood. Therefore, we analyzed six different points in time after 60 min of ischemia (at 140 mmHg): 2 h, 6 h, 12 h, 24 h, 3 days, and 7 days. We demonstrated an impaired functionality of the INL and photoreceptors, via ERG measurements, as well as a progressive loss of amacrine, cone bipolar and cone photoreceptor cells, via immunohistology and qRT-PCR. Also, RGCs are very sensitive to ischemia. The number of this cell type decreased progressively over time. Moreover, an increased expression of macroglia was detected, starting as early as 2 h post I/R.

## Materials and Methods

### Animals

Male Brown-Norway rats (7–8 weeks old; Charles River Laboratories, Sulzfeld, Germany) were used for the analyses. The study was approved by the animal care committee of North Rhine-Westphalia (Germany), all experiments were carried out in accordance with the ARVO statement for the use of animals in ophthalmic and vision research. Rats were housed under environmentally controlled conditions (12-h light-dark cycle) with free access to chow and water.

### Induction of Ischemia/Reperfusion

Retinal I/R was induced as previously described (Schmid et al., 2014; Joachim et al., 2017; Palmhof et al., 2018). Animals were anesthetized with a ketamine/xylazine/vetranquil cocktail (0.65/0.65/0.2 ml; 1.5 ml/kg body weight). One eye per animal was dilated using 5% tropicamide (Pharma Stulln, Stulln, Germany) and anesthetized topically with conjuncain (Bausch & Lomb, Berlin, Germany). In addition, metamizol (100 mg/kg; Zentiva, Frankfurt am Main, Germany), a non-opioid analgesic drug, was administered subcutaneously. By elevating a saline reservoir containing 0.9% NaCl (Fresenius SE & Co. KGaA, Bad Homburg, Germany), the IOP was raised to 140 mmHg for 60 min. The saline reservoir was connected to a 27-gauge needle (Terumo Europe, Leuven, Belgium), which was placed into the anterior chamber of one eye. Retinal ischemia was confirmed by observing whitening of the retina and reperfusion was reassured by observing the returning blood flow with an ophthalmoscope (Mini 300; Heine Optotechnik, Herrsching, Germany). The contralateral eye remained untreated and served as control.

### Electroretinogram Measurements

Retinal function was monitored using full-field flash electroretinography (HMsERG system; OcuScience LLC, Rolla, MO, United States). The measurements were performed 3 (*n* = 9/group) and 7 days (*n* = 10/group) after induction of I/R. Therefore, rats were first dark adapted under dim red light as previously described (Palmhof et al., 2018). After anesthesia of the animals with a ketamine/xylazine cocktail (100/4 mg/kg), the eyes were dilated with 5% tropicamide and topically anesthetized with conjuncain. Reference electrodes were placed subcutaneously below the right and left ear and a ground electrode was placed in the base of the tail. After application of methocel (Omni Vision, Puchheim, Germany), contact lenses including silver thread recording electrodes were attached central on the cornea. Scotopic flash ERGs were recorded at 0.1, 0.3, 1, 3, 10, and 25 cd.s/m^2^. At the light intensities of 0.1–3 candela (cd) four measurements were taken per light intensity, respectively. At 10 and 25 cd one measurement was performed. The light intensity was increased 60 s after the previous light stimulus, respectively. Regarding the light intensities of 0.1–3 cd, the waiting period (inter-stimulus interval) between the individual light stimuli within one light intensity lasted 10 s. Signals obtained from the corneal surface were amplified, digitized, averaged, and stored using commercial software (ERGView 4.380R; OcuScience LLC) for later analysis. A 150 Hz filtering of the data was applied before evaluating the a- and b-wave amplitudes. After a transfer of the data to a spreadsheet program (Excel; Microsoft Corp., Redmond, WA, United States), statistical analysis followed (Statistica V12; Statsoft, Tulsa, OK, United States).

### Tissue Collection and Processing

At all points in time (2 h, 6 h, 12 h, 24 h, 3 days, and 7 days after I/R) the eyes were removed and processed for (immuno-) histology (*n* = 7–8/group) and qRT-PCR (*n* = 5/group). For (immuno-) histology, the eye balls were fixed in 4% paraformaldehyde, incubated in 30% sucrose, embedded in optical cutting temperature medium (Tissue-Tek; Thermo Fisher Scientific, Cheshire, United Kingdom), and stored at -80°C. With a cryostat (Thermo Fisher Scientific, Walldorf, Germany), 10 μm thick retinal cross-sections were prepared for further stainings. For qRT-PCR analyses, the retina was dissected out, snap frozen in a lysis buffer with β-mercaptoethanol (Sigma-Aldrich, Steinheim, Germany) in liquid nitrogen, and stored at -80°C until RNA extraction.

### Retinal Histology

Three sections per eye were stained with H&E to obtain a structural overview of the retinal layers (*n* = 7–8/group/point in time). After the H&E staining, all slides were dehydrated in ethanol following incubation in xylene before being mounted with Eukitt (O. Kindler GmbH & Co, Freiburg, Germany). Two pictures per H&E stained retinal cross-section were taken at a distance of 1,500 μm dorsal and ventral to the optic nerve with a microscope equipped with a CCD camera (Axio Imager M1, Carl Zeiss Microscopy). The thickness of the whole retina (excluding the outer segment) and retinal layers (GCL, IPL, INL, OPL, ONL) was analyzed via a measuring tool in the Zen 2012 software (Zeiss) (Horstmann et al., 2013). For each analysis, three measurements per picture were prepared and then averaged.

### Immunohistology of Retinal Sections

Retinal cross-sections (*n* = 7–8/group/point in time) were also used for immunohistochemistry, as described previously (Reinehr et al., 2016; Palmhof et al., 2018). Therefore, the sections were first dried and rehydrated in PBS, followed by blocking in 10–20% appropriate serum with or without 1% BSA in 0.1% Triton X-100 in PBS. Six retinal sections per eye were used for each staining. RGCs, AII amacrine cells, cone as well as rod bipolar cells, macroglia, and cone as well as rod photoreceptor cells were investigated using specific antibodies (Table 1). Additionally, DAPI (4′,6-Diamidin-2-phenylindol; Serva Electrophoresis, Heidelberg, Germany) was applied as a nuclear stain. For each staining, negative controls were performed by utilizing only the secondary antibody. Four pictures per retinal cross-section, two from each periphery and two from the central part, were taken with a fluorescence microscope as described previously (Axio Imager M1 and M2; Carl Zeiss Microscopy) (Schmid et al., 2014; Joachim et al., 2017). The pictures were taken at a distance of approximately 300 and 3,100 μm dorsal and ventral to the optic nerve. All digitalized images were transferred to Corel Paint Shop Photo Pro (V 13; Corel Corp., Fremont, CA, United States), masked, and equal excerpts were cut out of each picture, which were used for the evaluation. The cut outs were prepared of a defined area of the retina with a total size of 800 pixel × 600 pixel (125.14 μm × 93.86 μm).

**Table 1:** List of used primary and secondary antibodies, including cell type, dilution, and company.

Primary antibody	Cell type	Species clonality, type	Dilution	Company and order number	Secondary antibody	Dilution	Company
Brn-3a	Retinal ganglion cells (RGCs)	Goat, polyclonal, IgG	1:100	Santa Cruz Biotechnology, Heidelberg, Germany (sc-31984)	Donkey anti-goat Alexa Fluor 488	1:500	Dianova, Hamburg, Germany
Cone arrestin	Photoreceptor cones	Rabbit, polyclonal, IgG	1:500	Millipore, Darmstadt, Germany (AB15282)	Donkey anti-rabbit Alexa Fluor 555	1:500	Invitrogen, Darmstadt, Germany
GFAP	Macroglia	Chicken, polyclonal, IgG	1:1000	Millipore (AB5541)	Donkey anti-chicken Cy3	1:700	Millipore
Opsin (red/green)	Photoreceptor cones	Rabbit, polyclonal, IgG	1:2000	Millipore (AB5405)	Donkey anti-rabbit Alexa Fluor 555	1:600	Invitrogen
Parvalbumin	AII amacrine cells	Goat, polyclonal, IgG	1:100	Swant, Marly, Switzerland (PVG-213)	Donkey anti-goat Alexa Fluor 488	1:500	Invitrogen
PKCα	Rod bipolar cells	Mouse, monoclonal, IgG	1:500	Santa Cruz Biotechnology (sc-8393)	Goat anti-mouse Alexa Fluor 488	1:500	Invitrogen
Recoverin	Cone bipolar cells	Rabbit, polyclonal, IgG	1:1000	Millipore (AB5585)	Donkey anti-rabbit Alexa Fluor 555	1:400	Invitrogen
Rhodopsin	Photoreceptor rods	Mouse, monoclonal, IgG	1:400	Abcam, Cambridge, United Kingdom (ab3267)	Goat anti-mouse Alexa Fluor 488	1:500	Invitrogen

Evaluation was carried out under masked conditions with ImageJ software (V 1.44p; NIH, Bethesda, MD, United States). The Brn-3a^+^, cone arrestin^+^, parvalbumin^+^, PKCα^+^, recoverin^+^, and opsin^+^ cells were counted and averaged for each eye (Schmid et al., 2014). For analysis of the GFAP and rhodopsin staining, the images were transferred to ImageJ, where they first were transformed into gray scale. After subtraction of the background (GFAP: 50 pixel; rhodopsin: 78.5 pixel), the lower (GFAP: 5.2; rhodopsin: 4.75) and upper thresholds (GFAP: 255; rhodopsin: 258.46) were set. Background subtraction and lower and upper threshold represent mean values of both groups. For each picture, the percentage of the GFAP^+^ and rhodopsin^+^ labeled area was measured using an ImageJ macro.

### Quantitative Real-Time PCR Analysis of Retinal Tissue

Total RNA from retinal tissue (*n* = 5/group/point in time) was extracted and purified according to the manufacturer’s instructions using the Gen Elute Mammalian Total RNA Miniprep Kit (Sigma-Aldrich). RNA concentration and purity were determined via spectrophotometry (BioSpectrometer; Eppendorf, Hamburg, Germany). To receive cDNA, 1 μg of total RNA was reverse-transcribed with a cDNA-synthesis kit (First Strand cDNA Synthesis Kit; Thermo Fisher Scientific, Waltham, MA, United States) and random hexamer primers (Sigma-Aldrich). A PikoReal 96 Real-Time PCR System (Thermo Fisher Scientific) with SYBR Green (DyNAmo Flash SYBR Green qPCR Kit; Thermo Fisher Scientific) was used to perform qRT-PCR experiments. Primer efficiencies of each primer set were calculated based on a dilution series of 5–125 ng cDNA (Joachim et al., 2017). The relative *Gfap*, *Opn1mw*, *Opn1sw*, *Pou4f1*, *Recoverin*, and *Rhodopsin* mRNA expression was evaluated. For normalization and relative quantification, *C*t values of the house-keeping genes *Actin* and *Cyclophilin* were consulted (Table 2).

**Table 2:** List of primer pairs used for analyses of RGCs, cells of the INL, and photoreceptor mRNA expression in control and ischemic retinae by qRT-PCR.

Gene	Primer sequence	Amplicon size	Primer efficiency
*β-Actin-F* *β-Actin-R*	cccgcgagtacaaccttct cgtcatccatggcgaact	72 bp	1.000
*Cyclophilin-F* *Cyclophilin-R*	tgctggaccaaacacaaatg cttcccaaagaccacatgct	88 bp	1.000
*Brn-3a (Pou4f1)-F* *Brn-3a (Pou4f1)-R*	ctggccaacctcaagatcc cgtgagcgactcgaacct	72 bp	0.732
*Gfap-F* *Gfap-R*	tttctccaacctccagatcc gaggtggccttctgacacag	64 bp	0.875
*Opn1mw-F* *Opn1mw-R*	tcatcgtgctctgctacctc tctttctgttgctttgccact	64 bp	1.000
*Opn1sw-F* *Opn1sw-R*	ccccatcatctactgcttcat agccagacatgtcagattcgt	98 bp	1.000
*Recoverin-F* *Recoverin-R*	aagatctgggcgtcctttg agggtcccctcgatgaat	71 bp	1.000
*Rhodopsin-F* *Rhodopsin-R*	accttgagggcttctttgc tcaatggccaggactacca	70 bp	1.000

*For relative quantification of mRNA levels, the house-keeping genes Actin and Cyclophilin were used. The primer sequence, the predicted amplicon size and the primer efficiency are indicated. bp, base pairs; F, forward; R, reverse.*

### Statistics

ERG and (immuno-) histological data are presented as mean ± SEM and qRT-PCR data as median ± quartile + minimum + maximum. At the (immuno-) histological analyses the controls were always set to 100%. Regarding statistic of electroretinography and histology, both groups, at each point in time, were compared using Student’s *t*-test (Statistica V13; Dell, Tulsa, OK, United States). For statistical evaluation of relative expression variations in qRT-PCR analyses, data were analyzed by REST^©^ software (QIAGEN GmbH, Hilden, Germany) using a pairwise fixed reallocation and randomization test. *P*-values below 0.05 were considered statistically significant.

## Results

**FIGURE 1:**
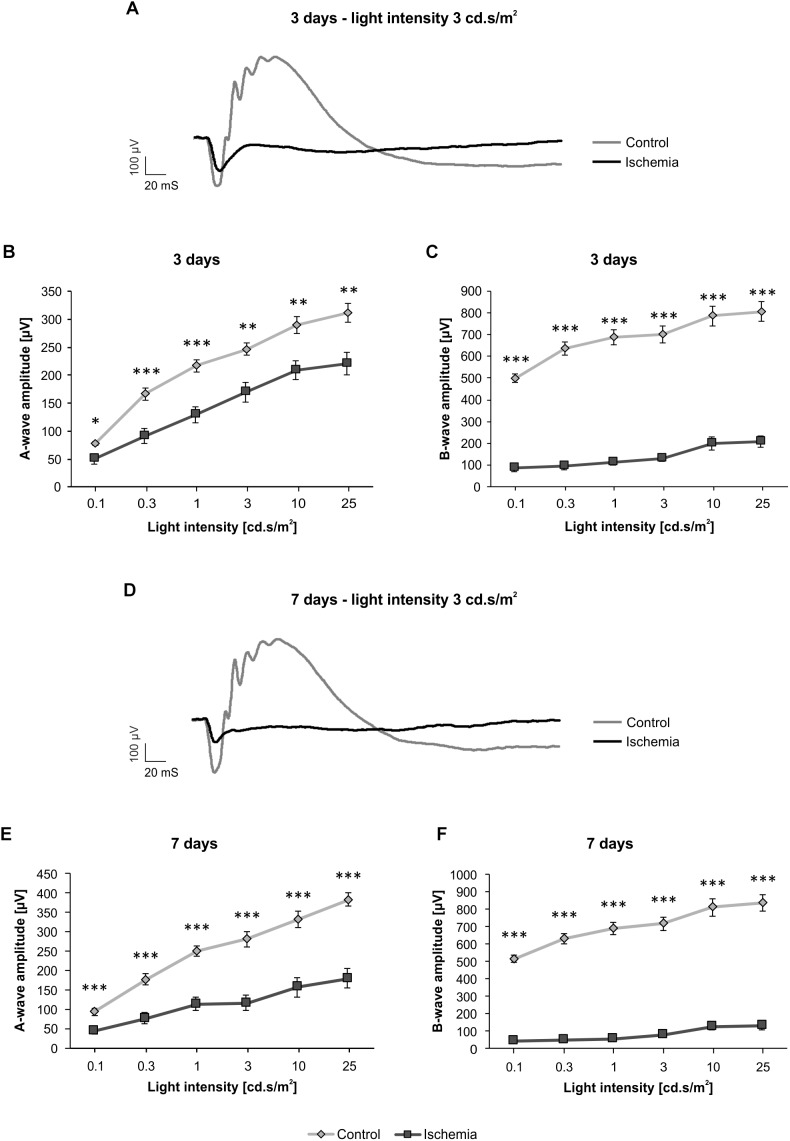
Electroretinogram (ERG) measurements were performed 3 (*n* = 9/group) and 7 (*n* = 10/group) days after ischemia induction. **(A)** Representative ERG recording 3 days after ischemia induction. The average graph of the obtained waveforms of control eyes and the contralateral ischemic eyes at 3 cd.s/m^2^ is shown. **(B)** A significant decrease of the a-wave amplitude (*p* < 0.05) was noted in ischemic eyes compared to control ones 3 days after I/R at all measured light intensities. **(C)** Additionally, a significantly lower b-wave amplitude (*p* < 0.001) was revealed in the ischemic group at this point in time. **(D)** Representative ERG recording 7 days after I/R. The average graph of the obtained waveforms of control eyes and the contralateral ischemic eyes at 3 cd.s/m^2^ is represented. **(E)** 7 days after ischemia induction, the significant reduction of the a-wave amplitude (*p* < 0.001) was still prominent and more pronounced. **(F)** A similar effect could be observed regarding the b-wave amplitude (*p* < 0.001) at this point in time. ^∗^*p* < 0.05, ^∗∗^*p* < 0.01, ^∗∗∗^*p* < 0.001.

The ERG measurements 3 days after I/R showed a significant decrease of the a-wave amplitude in ischemic eyes in comparison to the control group (*p* < 0.05; Figure 1B and Table 3A). The analyses of this amplitude display the photoreceptor activity. Also, the amplitude of the b-wave (*p* < 0.001), which reflects the cell activity of the INL, was significantly reduced after ischemia (Figure 1C and Table 3A). At this point in time, the reduction of the b-wave amplitude was more intensive than the one of the a-wave (Figure 1A). This effect of ischemia induction was still prominent after 7 days (Figure 1D). Also, at this later point in time, a significant diminution of the a-wave amplitude was revealed after ischemic injury (*p* < 0.001; Figure 1E and Table 3B). Compared to day 3, a stronger ischemic-induced decrease was noticed at all light intensities. The same observations were made for the b-wave amplitude. A significant decline of the b-wave amplitude was measured in ischemic eyes at all light intensities after 7 days (*p* < 0.001; Figure 1F and Table 3B).

**Table 3A:** Analyses of the a- and b-wave amplitude of the ERG measurements (mean ± SEM) at 3 days.

3 days						
**Light intensity [cd.s/m^2^]**	**0.1**	**0.3**	**1**	**3**	**10**	**25**
**A-wave amplitude [μm]**						
Control	77.7 ± 3.5	166.3 ± 10.5	216.7 ± 10.9	246.7 ± 11.1	289.9 ± 15.1	311.4 ± 16.7
Ischemia	50.3 ± 9.0	91.4 ± 13.7	129.9 ± 14.3	169.9 ± 17.2	208.9 ± 17.3	221.1 ± 19.7
*P*-value	0.012	0.0005	0.0002	0.002	0.003	0.003
**B-wave amplitude [μm]**						
Control	499.2 ± 18.8	635.6 ± 29.1	687.0 ± 35.0	700.5 ± 37.7	785.1 ± 45.0	806.3 ± 43.6
Ischemia	87.7 ± 17.1	96.5 ± 17.9	113.4 ± 14.7	130.9 ± 14.3	200.6 ± 30.3	208.3 ± 25.4
*P*-value	<0.001	<0.001	<0.001	<0.001	<0.001	<0.001

*Significant values are marked in red.*

**Table 3B:** Analyses of the a-and b-wave amplitude of the ERG measurements (mean ± SEM) at 7 days.

7 days						
**Light intensity [cd.s/m^2^]**	**0.1**	**0.3**	**1**	**3**	**10**	**25**
**A-wave amplitude [μm]**						
Control	93.7 ± 8.7	177.0 ± 14.3	249.7 ± 12.9	280.8 ± 18.9	333.2 ± 21.0	383.7 ± 17.4
Ischemia	45.4 ± 8.0	76.5 ± 14.9	113.9 ± 17.0	116.1 ± 20.4	156.5 ± 25.6	179.6 ± 25.0
*P*-value	0.0007	0.0001	<0.001	<0.001	<0.001	<0.001
**B-wave amplitude [μm]**						
Control	515.8 ± 19.3	630.0 ± 29.9	689.6 ± 37.3	718.4 ± 38.7	811.4 ± 48.8	837.4 ± 48.4
Ischemia	43.6 ± 10.1	49.1 ± 9.9	55.9 ± 7.9	79.0 ± 11.0	127.4 ± 18.8	130.8 ± 21.1
*P*-value	<0.001	<0.001	<0.001	<0.001	<0.001	<0.001

*Significant values are marked in red.*

**FIGURE 2:**
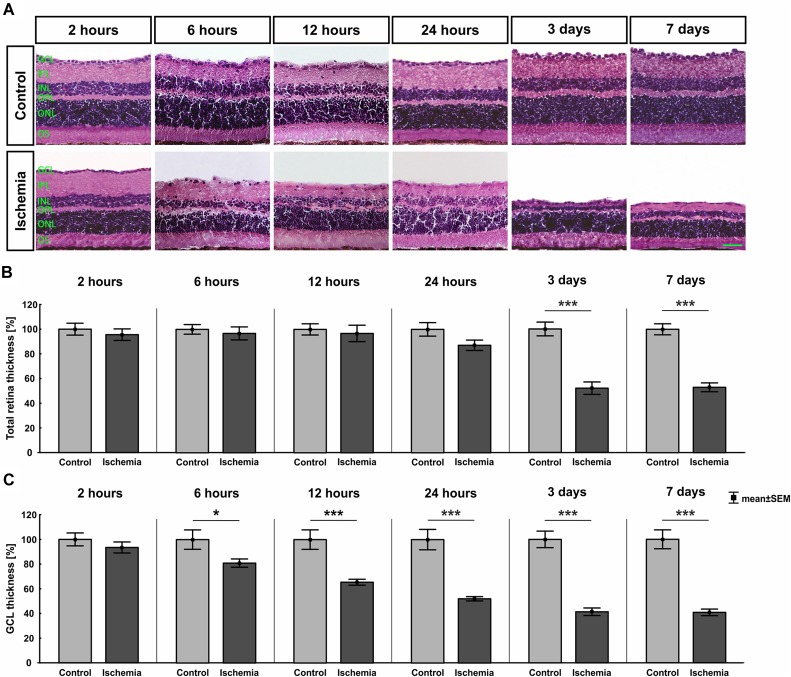
**(A)** Retinal cross-sections of all points in time were stained with H&E (*n* = 7–8/group). A decline of the total retinal thickness was observed in the ischemia group over time. **(B)** A significant decrease of the whole retinal thickness was measured in ischemic eyes starting at day 3 after I/R (3 days, 7 days: *p* < 0.001). **(C)** The GCL seems to be most sensitive against an ischemic insult. The thickness of this layer was significantly reduced already at 6 h (*p* = 0.036). This degeneration progressed over time (12 h: *p* = 0.0009; 24 h, 3 days, 7 days: *p* < 0.001). ^∗^*p* < 0.05, ^∗∗∗^*p* < 0.001. GCL, ganglion cell layer; IPL, inner plexiform layer; INL, inner nuclear layer; OPL, outer plexiform layer; ONL, outer nuclear layer; OS, outer segment. Scale bar: 20 μm.

The H&E stained retinal cross-sections were utilized to measure the thickness of the different retinal layers. A distinct degradation of the whole retina started on day 3 after I/R (Figure 2A). This observation was confirmed by the measurements. A significant reduced thickness of the whole retina was detected at day 3 (*p* < 0.001) and 7 (*p* < 0.001) in ischemic eyes when compared to the control group (Figure 2B and Table 4). Regarding the GCL alone, an ischemic-induced damage was established at an earlier point in time. A significantly thinner GCL in ischemic eyes was already noticeable after 6 h (*p* = 0.036; Figure 2C and Table 4). This difference was still evident at 12 h (*p* = 0.0009), 24 h (*p* < 0.001), 3 days (*p* < 0.001), and 7 days (*p* < 0.001) in comparison to control retinae. Also, changes in the layer thickness in the other retinal layers (IPL, INL, OPL, and ONL) were measured after ischemia induction. Here, the layer thicknesses were reduced mainly at 3 and 7 days (*p* < 0.001; Table 4). However, the OPL also seems to be sensitive to an ischemic injury. A decrease of this layer was already present 6 h after ischemia (*p* = 0.001; Table 4).

**Table 4:** Analyses of the layer thicknesses (%) of the H&E stained cross-sections at all points in time.

H&E [%]						
**Points in time**	**2 h**	**6 h**	**12 h**	**24 h**	**3 days**	**7 days**
**Total retina**						
Control	100.0 ± 4.8	100.0 ± 3.9	100.0 ± 4.5	100.0 ± 5.5	100.0 ± 5.6	100.0 ± 4.5
Ischemia	95.6 ± 4.7	96.8 ± 5.3	96.7 ± 6.7	87.1 ± 4.2	52.0 ± 5.0	53.0 ± 3.6
*P*-value	0.518	0.636	0.691	0.084	<0.001	<0.001
**GCL**						
Control	100.0 ± 5.2	100.0 ± 7.9	100.0 ± 7.9	100.0 ± 8.3	100.0 ± 6.7	100.0 ± 7.7
Ischemia	93.4 ± 4.5	81.0 ± 3.4	65.5 ± 2.4	52.1 ± 1.7	41.4 ± 3.1	40.8 ± 2.7
*P*-value	0.357	0.036	0.0009	<0.001	<0.001	<0.001
**IPL**						
Control	100.0 ± 7.4	100.0 ± 4.6	100.0 ± 9.0	100.0 ± 7.5	100.0 ± 8.2	100.0 ± 6.3
Ischemia	97.1 ± 6.6	101.9 ± 9.7	97.9 ± 9.3	79.5 ± 6.5	22.8 ± 2.8	21.6 ± 2.1
*P*-value	0.772	0.873	0.876	0.058	<0.001	<0.001
**INL**						
Control	100.0 ± 4.4	100.0 ± 6.1	100.0 ± 5.3	100.0 ± 5.2	100.0 ± 4.6	100.0 ± 3.8
Ischemia	95.3 ± 4.1	88.7 ± 6.0	78.6 ± 4.4	88.9 ± 6.0	48.4 ± 6.4	45.4 ± 3.5
*P*-value	0.453	0.210	0.008	0.183	<0.001	<0.001
**OPL**						
Control	100.0 ± 3.4	100.0 ± 2.7	100.0 ± 5.0	100.0 ± 6.4	100.0 ± 10.6	100.0 ± 4.7
Ischemia	91.7 ± 3.4	84.6 ± 2.6	77.4 ± 4.1	78.2 ± 3.7	43.7 ± 5.8	58.5 ± 3.7
*P*-value	0.104	0.001	0.003	0.010	0.0004	<0.001
**ONL**						
Control	100.0 ± 3.1	100.0 ± 4.0	100.0 ± 4.7	100.0 ± 4.5	100.0 ± 3.4	100.0 ± 4.0
Ischemia	95.4 ± 3.6	89.4 ± 3.8	90.8 ± 4.2	92.1 ± 3.1	66.2 ± 4.0	74.4 ± 4.0
*P*-value	0.351	0.077	0.165	0.168	<0.001	0.0004

*Control groups were set at 100%. Significant values are marked in red. GCL, ganglion cell layer; IPL, inner plexiform layer; INL, inner nuclear layer; OPL, outer plexiform layer; ONL, outer nuclear layer.*

**FIGURE 3:**
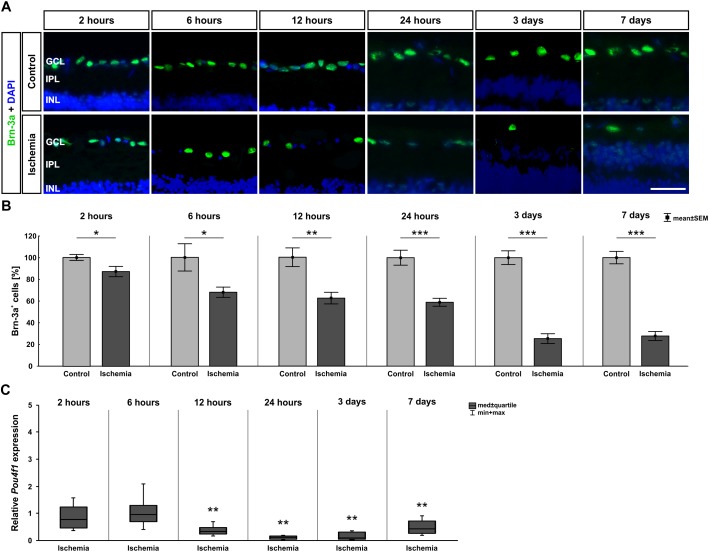
**(A)** Brn-3a (green) was used to stain RGCs on retinae of all points in time (*n* = 7–8/group). Cell nuclei were labeled with DAPI (blue). Fewer Brn-3a^+^ cells were observed in the ischemia group over time. **(B)** A significant RGC loss was noted 2 h after I/R (*p* = 0.032), which remained until day 7 and became increasingly stronger over the time (6 h: *p* = 0.026; 12 h: *p* = 0.002; 24 h, 3 days, 7 days: *p* < 0.001). **(C)** Via qRT-PCR, a significant down-regulation of *Pou4f1* mRNA levels was verified starting 12 h after ischemia (12 h: *p* = 0.003; 24 h: *p* = 0.007; 3 days: *p* = 0.006; 7 days: *p* = 0.001). ^∗^*p* < 0.05, ^∗∗^*p* < 0.01, ^∗∗∗^*p* < 0.001. GCL, ganglion cell layer; IPL, inner plexiform layer; INL, inner nuclear layer. Scale bar: 20 μm.

Since the GCL showed such an early and strong degeneration via H&E staining, we then focused on RGCs at the different points in time. Therefore, the specific ganglion cell marker anti-Brn-3a was applied on the sections. A progressive regression of RGCs was observed over time (Figure 3A). Statistical analyses of the immunohistological staining validated this impression. Significantly fewer Brn-3a^+^ ganglion cells were detected in the ischemic retinae starting after 2 h (*p* = 0.032). This RGC loss was prominent until day 7 (6 h: *p* = 0.026; 12 h: *p* = 0.002; 24 h, 3 days, 7 days: *p* < 0.001; Figure 3B and Table 5). Over time, a progressive course was established with an obvious decrease at day 3 (Figure 3B and Table 5). In addition, qRT-PCR analyses were performed to evaluate the *Brn-3a* (*Pou4f1*) expression on mRNA level. Compared to control retinae, a significant down-regulation of the relative *Brn-3a* expression was detected from 12 h (*p* = 0.003) on (Figure 3C and Table 5). The expression of *Brn-3a* mRNA remained diminished until day 7 (24 h: *p* = 0.007; 3 days: *p* = 0.006; 7 days: *p* = 0.001; Figure 3C and Table 5).

**Table 5:** Analyses of the Brn-3a staining (%), control groups were all set at 100%.

Brn-3a						
**Points in time**	**2 h**	**6 h**	**12 h**	**24 h**	**3 days**	**7 days**
**Immunohistology [cells in %]**						
Control	100.0 ± 2.7	100.0 ± 12.5	100.0 ± 8.6	100.0 ± 6.9	100.0 ± 6.3	100.0 ± 5.7
Ischemia	87.1 ± 4.7	68.0 ± 4.7	62.4 ± 5.3	58.9 ± 3.7	25.4 ± 4.5	27.7 ± 4.2
*P*-value	0.032	0.026	0.002	<0.001	<0.001	<0.001
**qRT-PCR [fold expression]**						
Relative expression	0.751	0.948	0.318	0.107	0.097	0.42
*P*-value	0.281	0.693	0.003	0.007	0.006	0.001

*Relative Pou4f1 mRNA expression (med) at all points in time are also shown. Significant values are marked in red.*

**FIGURE 4:**
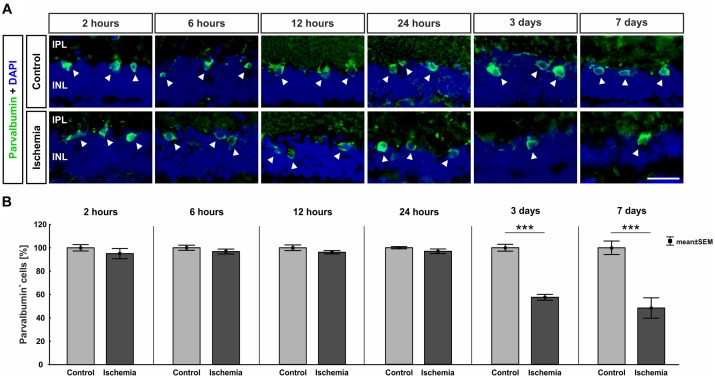
**(A)** AII amacrine cells were marked with an anti-parvalbumin antibody (green, arrows), while DAPI was used to label cell nuclei (blue; *n* = 7–8/group). A lower number of parvalbumin^+^ amacrine cells was noted at the later points in time. **(B)** A significant regression of parvalbumin^+^ cells was detected 3 (*p* < 0.001) and 7 days (*p* < 0.001) after ischemia. ^∗∗∗^*p* < 0.001. IPL, inner plexiform layer; INL, inner nuclear layer. Scale bar: 20 μm.

The ERG measurements showed a functional disorder of the cells in the INL 3 and 7 days after ischemia induction. Thus, we examined those cells in the next step. AII amacrine cells were labeled using anti-parvalbumin. While a similar number of parvalbumin^+^ cells was present in both groups at the early points in time (2–24 h), fewer parvalbumin^+^ amacrine cells were noted in the ischemic group at later points in time (3 and 7 days; Figure 4A). This observation was underpinned by statistical analyses. At 3 (*p* < 0.001) and 7 days (*p* < 0.001), ischemic retinae displayed a significant loss of parvalbumin^+^ cells (Figure 4B and Table 6A).

**Table 6A:** Analyses of the parvalbumin staining (%) at all points in time.

Parvalbumin						
**Points in time**	**2 h**	**6 h**	**12 h**	**24 h**	**3 days**	**7 days**
**Immunohistology [cells in %]**						
Control	100.0 ± 2.7	100.0 ± 2.2	100.0 ± 2.4	100.0 ± 1.0	100.0 ± 2.9	100.0 ± 5.8
Ischemia	95.0 ± 4.4	96.7 ± 2.1	96.2 ± 1.4	96.9 ± 2.0	57.5 ± 2.5	48.4 ± 8.7
*P*-value	0.349	0.306	0.195	0.186	<0.001	<0.001

*Control groups were set at 100%. Significant values are marked in red.*

**FIGURE 5:**
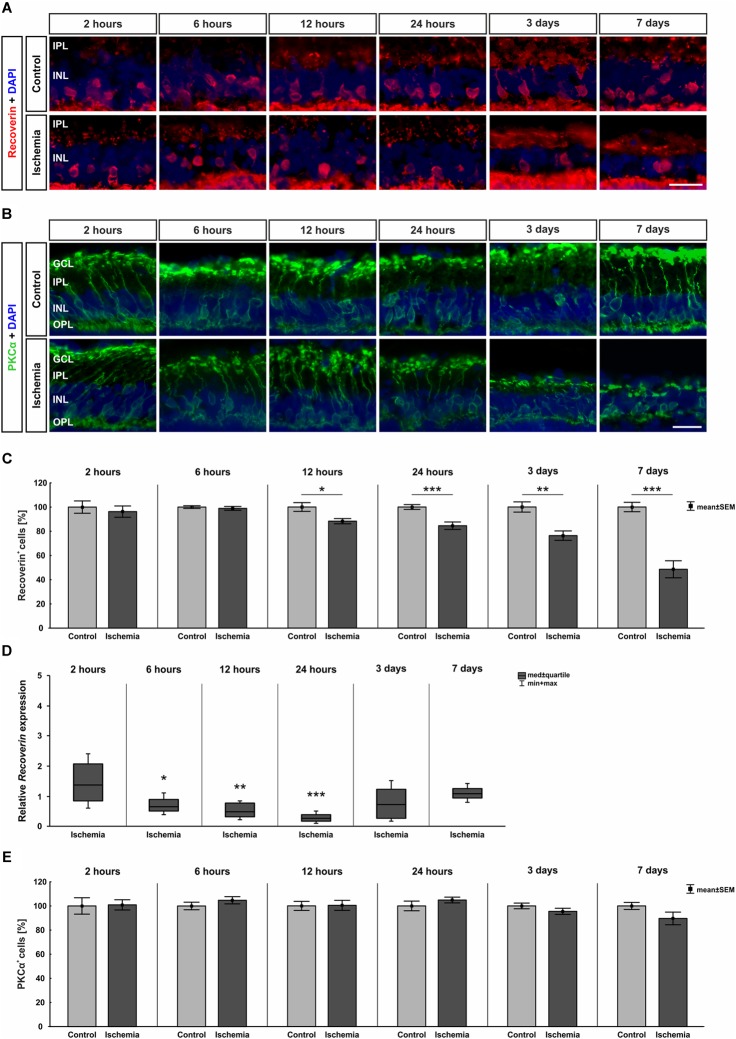
**(A)** Cone bipolar cells were stained with anti-recoverin (red) and cell nuclei with DAPI (blue; *n* = 7–8/group). After ischemia induction, fewer cells were observed over time. **(B)** Anti-PKCα was used to detect rod bipolar cells (green) and DAPI for cell nuclei (blue; *n* = 7–8/group). The cells were equally stained in both groups. **(C)** Counting of cone bipolar cells displayed a significant reduction of recoverin^+^ cells at 12 h (*p* = 0.015), 24 h (*p* < 0.001), 3 days (*p* = 0.001), and 7 days (*p* < 0.001) after ischemia induction. **(D)** On mRNA level, a significant down-regulation of *Recoverin* mRNA expression was shown in the ischemia group at 6 h (*p* = 0.024), 12 h (*p* = 0.006), and 24 h (*p* < 0.001). **(E)** Evaluation of rod bipolar cells revealed no differences in cell number between both groups and all points in time. ^∗^*p* < 0.05, ^∗∗^*p* < 0.01, ^∗∗∗^*p* < 0.001. GCL, ganglion cell layer; IPL, inner plexiform layer; INL, inner nuclear layer; OPL, outer plexiform layer. Scale bars: 20 μm.

Bipolar cells, especially cone bipolar cells, were detected using the marker anti-recoverin. Anti-PKCα was used to visualize rod bipolar cells. The immunolabeling showed a progressive diminution of recoverin^+^ cells over time, while the number of rod bipolar cells remained unchanged between both groups and all points in time (Figure 5A,B). Evaluating the staining of bipolar cones revealed a significant lower number of recoverin^+^ cells in ischemic eyes at 12 h (*p* = 0.015), 24 h (*p* < 0.001), 3 days (*p* = 0.001), and 7 days (*p* < 0.001) when compared to the control group (Figure 5C and Table 6B). In contrast, cell counts of rod bipolar cells indicated no differences in the cell number of PKCα^+^ cells between control and ischemic retinae at all points in time (2 h, 6 h, 12 h, 24 h, 3 days, 7 days: *p* > 0.05; Figure 5E and Table 6C). The relative expression level of *Recoverin* mRNA was investigated via qRT-PCR. Comparison of all points in time showed a significant down-regulation of *Recoverin* mRNA expression at 6 h (*p* = 0.024), 12 h (*p* = 0.006), and 24 h (*p* < 0.001) after ischemia. However, at 3 (*p* = 0.515) and 7 days (*p* = 0.406) ischemic retinae displayed a similar level as control eyes (Figure 5D and Table 6B).

**Table 6B:** Evaluation of recoverin staining (%) as well as of expression levels of *Recoverin* mRNA (med) at all points in time.

Recoverin						
**Points in time**	**2 h**	**6 h**	**12 h**	**24 h**	**3 days**	**7 days**
**Immunohistology [cells in %]**						
Control	100.0 ± 5.1	100.0 ± 1.1	100.0 ± 3.6	100.0 ± 2.0	100.0 ± 4.2	100.0 ± 3.9
Ischemia	96.3 ± 4.7	98.9 ± 1.6	88.3 ± 2.2	84.5 ± 3.1	76.3 ± 3.9	48.6 ± 7.0
*P*-value	0.597	0.594	0.015	<0.001	0.001	<0.001
**qRT-PCR [fold expression]**						
Relative expression	1.377	0.656	0.474	0.273	0.727	1.076
*P*-value	0.169	0.024	0.006	<0.001	0.515	0.406

*Regarding staining, control groups were set at 100%. Significant values are marked in red.*

**Table 6C:** Evaluation of the PKCα staining (%) at all points in time.

PKCα						
**Points in time**	**2 h**	**6 h**	**12 h**	**24 h**	**3 days**	**7 days**
**Immunohistology [cells in %]**						
Control	100.0 ± 6.8	100.0 ± 3.1	100.0 ± 3.8	100.0 ± 4.0	100.0 ± 2.4	100.0 ± 2.9
Ischemia	100.9 ± 4.2	104.7 ± 3.0	100.5 ± 4.2	104.9 ± 2.4	95.5 ± 2.6	89.6 ± 5.2
*P*-value	0.912	0.296	0.938	0.314	0.218	0.106

*Control groups were set at 100%.*

**FIGURE 6:**
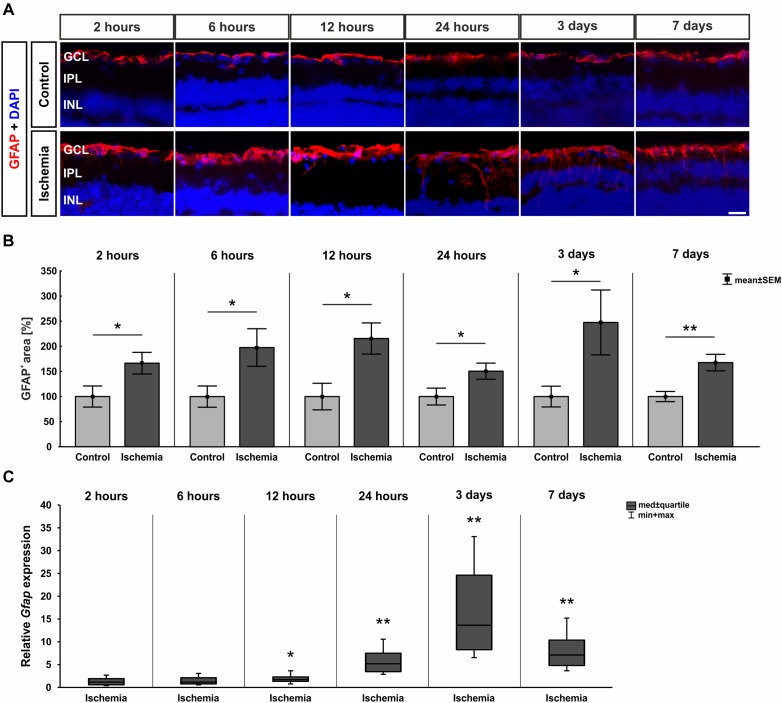
**(A)** Macroglia were detected with anti-GFAP (red), while DAPI was used for cell nuclei (blue; *n* = 7–8/group). Control retinae displayed GFAP in the GCL and NFL, while the signal spread to inner layers after ischemia. **(B)** An increase of GFAP^+^ area was measured after I/R for all points in time (2 h: *p* = 0.044; 6 h: *p* = 0.048; 12 h: *p* = 0.013; 24 h: 0.047; 3 days: 0.047; 7 days: 0.004). **(C)** Compared to controls, *Gfap* mRNA level was significantly higher at 12 h (*p* = 0.037), 24 h (0.007), 3 days (*p* = 0.004), and 7 days (*p* = 0.003) after ischemia. ^∗^*p* < 0.05, ^∗∗^*p* < 0.01. GCL, ganglion cell layer; IPL, inner plexiform layer; INL, inner nuclear layer. Scale bar: 20 μm.

In order to evaluate macroglia, the expression of GFAP was investigated on protein as well as on mRNA level. GFAP is mainly expressed by astrocytes and Müller glia. Regarding immuno-reactivity, GFAP was present in controls of all six points in time in close proximity to the GCL. After I/R induction, an increase in positive signal area was observed with formation of processes (Figure 6A). Analyses of the immunohistological staining revealed a significantly larger GFAP^+^ signal area at all points in time after ischemia in comparison to control retinae (2 h: *p* = 0.044; 6 h: *p* = 0.048; 12 h: *p* = 0.013; 24 h: *p* = 0.047; 3 days: *p* = 0.047; 7 days: *p* = 0.004; Figure 6B and Table 7). In addition, a significant up-regulation of *Gfap* mRNA was noted in ischemic retinae at 12 and 24 h as well as 3 and 7 days (12 h: *p* = 0.037; 24 h: *p* = 0.007; 3 days: *p* = 0.004; 7 days: *p* = 0.003; Figure 6C and Table 7).

**Table 7:** Analyses of the GFAP area (%), where control groups were set at 100%.

GFAP						
**Points in time**	**2 h**	**6 h**	**12 h**	**24 h**	**3 days**	**7 days**
**Immunohistology [area fraction in %]**						
Control	100.0 ± 21.0	100.0 ± 21.1	100.0 ± 26.4	100.0 ± 16.8	100.0 ± 20.7	100.0 ± 10.1
Ischemia	166.4 ± 21.5	197.7 ± 37.4	215.6 ± 31.1	150.5 ± 16.0	247.6 ± 64.6	167.6 ± 16.5
*P*-value	0.044	0.048	0.013	0.047	0.047	0.004
**qRT-PCR [fold expression]**						
Relative expression	1.198	1.246	1.774	5.239	13.771	7.147
*P*-value	0.511	0.374	0.037	0.007	0.004	0.003

*Relative Gfap mRNA expression (med) at all points in time was also evaluated. Significant values are marked in red.*

**FIGURE 7:**
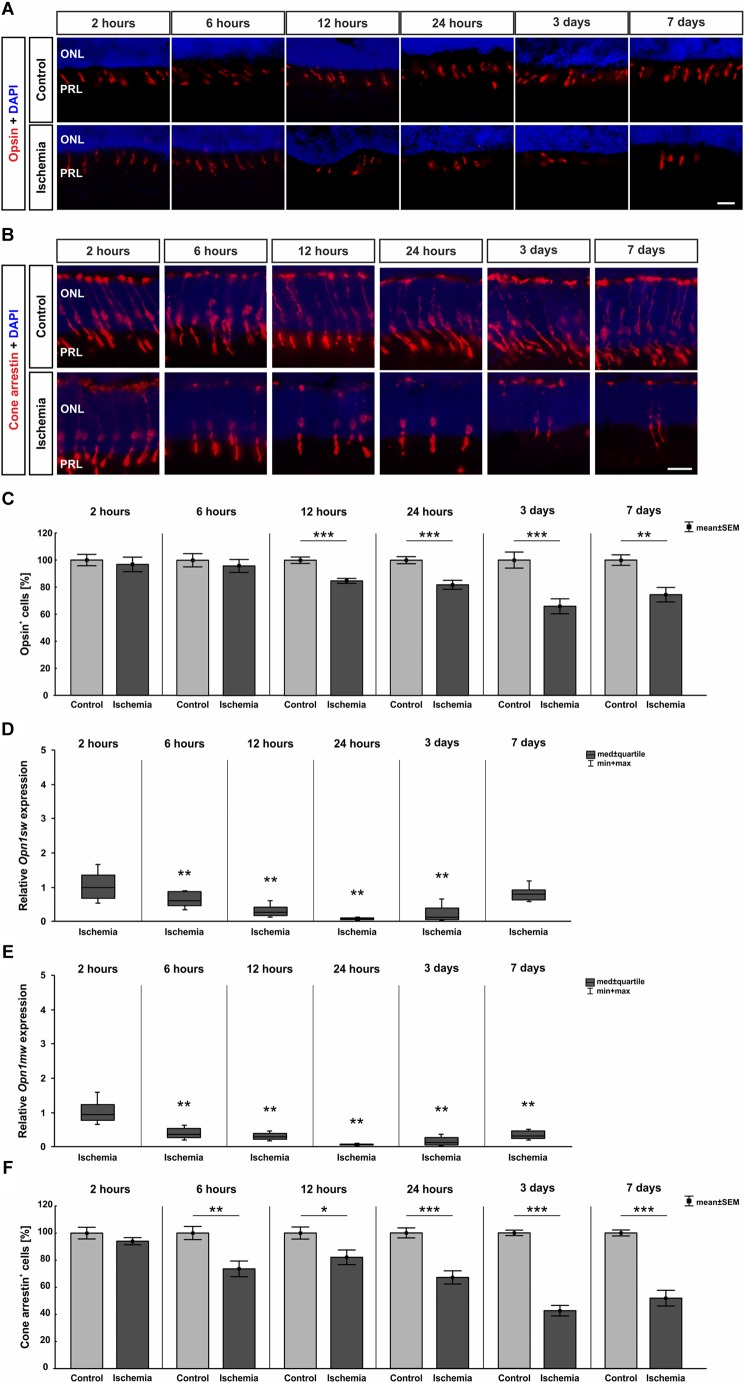
**(A)** Cone photoreceptor cells were visualized using anti-opsin (red) and cell nuclei (blue) using DAPI (*n* = 7–8/group). At 24 h, 3 days, and 7 days fewer opsin^+^ cells could be seen in retinae underlying I/R. **(B)** A second marker, anti-cone arrestin (red), was used to mark cones more specifically, while DAPI was again used for the cell nuclei (blue; *n* = 7–8/group). Fewer arrestin^+^ cell bodies were observed in the ONL of ischemic eyes from 6 h on **(C)**. Statistical analyses demonstrated a significant decreased number of opsin^+^ cells starting at 12 h (12 h, 24 h, 3 days: *p* < 0.001; 7 days: *p* = 0.002). **(D)** A down-regulation of *Opn1sw* mRNA expression was noted after ischemia at 6, 12, and 24 h and 3 days (all: *p* < 0.01), which was no longer significant on day 7. **(E)** Regarding *Opn1mw*, its mRNA expression was significantly decreased 6 h after I/R and stayed constantly low until day 7 (all: *p* < 0.01). **(F)** A reduction in number of cone arrestin^+^ cells was detected as early as 6 h after ischemia induction (6 h: *p* = 0.004; 12 h: *p* = 0.023; 24 h, 3 days, 7 days: *p* < 0.001). ^∗^*p* < 0.05, ^∗∗^*p* < 0.01, ^∗∗∗^*p* < 0.001. ONL, outer nuclear layer; PRL, photoreceptor layer. Scale bars: 20 μm.

Also, the activity of photoreceptors was impaired by I/R. Therefore, we investigated the outer retinal layer further. Antibodies specific to opsin as well as cone arrestin were used to visualize photoreceptor cones. While opsin^+^ cells were seen in the PRL, cone arrestin^+^ cell bodies were localized in the ONL. Fewer opsin^+^ and cone arrestin^+^ cells were detected in ischemic eyes at 12 h, 24 h, 3 days, and 7 days (Figure 7A,B). This observation was confirmed by statistical analyses. Two hours after ischemia, control (100.0 ± 4.2% 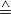 40.9 ± 1.7 cells/mm) and ischemic retinae (96.8 ± 5.4% 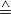 39.6 ± 2.2 cells/mm) had very similar numbers of opsin^+^ cells (*p* = 0.6). Opsin cell counts revealed a significant reduced cell number at 12 h (*p* = 0.0002), 24 h (*p* = 0.0007), and 3 days (*p* = 0.0009) after ischemia. At 7 days, only 74.4 ± 5.4% of opsin^+^ cells (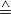 38.9 ± 2.8 cells/mm) were still present in the ischemia group in comparison to 100.0 ± 3.9% (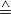 52.2 ± 2.0 cells/mm) in the control group (*p* = 0.002; Figure 7C and Table 8A). In regard to cone arrestin, the control group (100.0 ± 4.3% 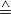 53.4 ± 2.3 cells/mm) and the ischemia group (94.1 ± 2.6% 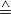 50.2 ± 1.4 cells/mm) displayed comparable cell numbers (*p* = 0.3). But absolute numbers for this cell type were a little bit higher than for opsin. Significantly fewer arrestin^+^ cones were counted from 6 h post I/R on in comparison to controls (6 h: *p* = 0.004; 12 h: *p* = 0.023; 24 h, 3 days: *p* < 0.001). At 7 days, 51.9 ± 5.8% arrestin^+^ cells (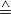 28.3 ± 3.2 cells/mm) were observed in the ischemia group in contrast to 100.0 ± 2.2% (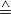 54.6 ± 1.2 cells/mm) in the control group (*p* < 0.001; Figure 7F and Table 8B). In order to differentiate between short-wavelength and medium-wavelength sensitive cone opsin we also evaluated the expression levels of *Opn1sw* (short-wavelength sensitive cone opsin) and *Opn1mw* (medium-wavelength sensitive cone opsin) mRNA. Regarding *Opn1sw* mRNA, a significantly reduced expression was noted 6 h after ischemia (*p* = 0.003). This down-regulation was also present after 12 h (*p* = 0.002), 24 h (*p* = 0.005), and 3 days (*p* = 0.009; Figure 7D and Table 8A). Although there was a trend, there were no significant differences in *Opn1sw* mRNA level detected anymore between ischemic and control retinae at day 7 (*p* = 0.058; Figure 7D and Table 8A). A significant decrease of *Opn1mw* mRNA level was measured in ischemic retinae from 6 h on (*p* = 0.002). This down-regulation was present throughout the study (12 h: *p* = 0.002; 24 h: *p* = 0.003; 3 days: *p* = 0.008; 7 days: *p* = 0.003; Figure 7E and Table 8A).

**Table 8A:** Analyses of the opsin staining (%) at all points in time.

Opsin						
**Points in time**	**2 h**	**6 h**	**12 h**	**24 h**	**3 days**	**7 days**
**Immunohistology [cells in %]**						
Control	100.0 ± 4.2	100.0 ± 4.9	100.0 ± 2.4	100.0 ± 2.6	100.0 ± 5.9	100.0 ± 3.9
Ischemia	96.8 ± 5.4	95.8 ± 4.8	84.7 ± 1.9	81.8 ± 3.3	65.9 ± 5.5	74.4 ± 5.4
*P*-value	0.646	0.552	0.0002	0.0007	0.0009	0.002
**qRT-PCR: *Opn1sw* [fold expression]**						
Relative expression	0.997	0.614	0.260	0.075	0.134	0.791
*P*-value	0.992	0.003	0.002	0.005	0.009	0.058
**qRT-PCR: *Opn1mw* [fold expression]**						
Relative expression	0.945	0.372	0.279	0.058	0.119	0.326
*P*-value	0.621	0.002	0.002	0.003	0.008	0.003

*Control groups were set at 100%. In addition, levels of Opn1sw and Opn1mw mRNA expression were evaluated. Significant values are marked in red.*

**Table 8B:** Analyses of the cone arrestin staining (%) at all points in time.

Cone arrestin						
**Points in time**	**2 h**	**6 h**	**12 h**	**24 h**	**3 days**	**7 days**
**Immunohistology [cells in %]**						
Control	100.0 ± 4.3	100.0 ± 4.9	100.0 ± 4.5	100.0 ± 3.7	100.0 ± 2.0	100.0 ± 2.2
Ischemia	94.1 ± 2.6	73.6 ± 5.8	82.1 ± 5.4	67.2 ± 4.9	42.6 ± 3.9	51.9 ± 5.8
*P*-value	0.258	0.004	0.023	<0.001	<0.001	<0.001

*Control groups were set at 100%. Significant values are marked in red.*

**FIGURE 8:**
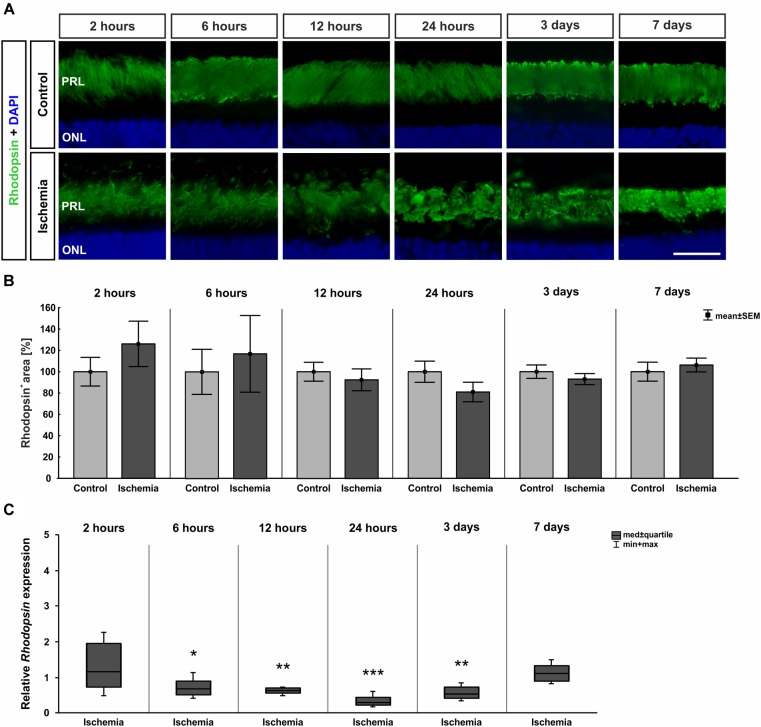
**(A)** Rod photoreceptor cells were stained with anti-rhodopsin (green; *n* = 7–8/group). DAPI marked cell nuclei (blue). While in control retinae the structure was well defined, a disorganization was noted after ischemia at later points in time. **(B)** Regarding the analyses of rhodopsin^+^ staining area, no differences were seen at all points in time. **(C)** Certainly, on mRNA level, a significant down-regulation of *Rhodopsin* mRNA expression could be shown in the ischemia group after 6 h (*p* = 0.026), 12 h (*p* = 0.007), 24 h (*p* < 0.001), and 3 days (*p* = 0.008). ^∗^*p* < 0.05, ^∗∗^*p* < 0.01, ^∗∗∗^*p* < 0.001. PRL, photoreceptor layer; ONL, outer nuclear layer. Scale bar: 20 μm.

Photoreceptor rods were labeled with anti-rhodopsin. From 12 h on, distinct changes concerning the rhodopsin structure were noted. A resolution and disorganization of the photoreceptor rod structure was observed on all ischemic retinae (Figure 8A). However, statistical analyses displayed no significant differences in rhodopsin^+^ staining area at any point in time between both groups (2 h: *p* = 0.317; 6 h: *p* = 0.703; 12 h: *p* = 0.584; 24 h: *p* = 0.181; 3 days: *p* = 0.407; 7 days: *p* = 0.579; Figure 8B and Table 8C). While the staining area was comparable over time, a significantly reduced expression of relative *Rhodopsin* mRNA was noted at 6 h (*p* = 0.026), 12 h (*p* = 0.007), 24 h (*p* < 0.001), and 3 days (*p* = 0.008) after ischemia via qRT-PCR (Figure 8C and Table 8C). However, no difference in *Rhodopsin* mRNA level was measured between ischemic and control retinae at day 7 (*p* = 0.26; Figure 8C and Table 8C).

## Discussion

Neurodegenerative diseases that affect the retina, like AMD, diabetic retinopathy, glaucoma, and retinal vascular occlusion, are still not properly treatable or even curable. This is due to the complexity of the underlying mechanisms of the progress of the diseases. It is known that ischemia is mainly involved in the degeneration of the retina (Cai et al., 2015). However, the impact of an ischemic injury on the retina and its cell types, especially over time, has not been fully explored and understood. Particularly, for the development of new therapy options and drug testing it is important to know how the ischemic damage spreads and evolves over time. Some research groups have investigated different points in time after ischemia induction and tried to create a timeline of retinal degeneration. However, in these studies, the researchers were focused only on the early or late points in time or solely inner retinal layers, like the GCL and INL (Unoki and LaVail, 1994; Weber et al., 1996; Dijk et al., 2004a,b; Slater et al., 2008; Kim et al., 2013; Zhao et al., 2013). For example, the group around Zhao et al. prepared analyses regarding retinal morphology at early points in time, from 6 until 48 h (Zhao et al., 2013). Sellés-Navarro et al. (1996) and Kim et al. (2013) examined morphological changes at later points in time (3–28 days and 5–30 days) with a focus on retinal thickness and/or RGC loss. There are also publications which demonstrated long-term analyses (e.g., 12 h–15 days, 1–31 days, or 2 h–7 days), but these studies focused only on specific factors, like the profile of the retinal transcriptome, the apoptosis of RGCs, or the behavior of the INL and its cell types, respectively (Dijk et al., 2004a,b; Agudo et al., 2008; Slater et al., 2008). Furthermore, different animal models were used in the mentioned studies to induce retinal damage, including transient global ischemia, (pressure-induced) retinal I/R, optic nerve transection, optic nerve crush, or anterior ischemic optic neuropathy (Dijk et al., 2004a; Agudo et al., 2008; Slater et al., 2008; Kim et al., 2013; Zhao et al., 2013). Accordingly, this has different ischemic effects on the degeneration of the retina and its cell types.

We used the pressure-induced I/R model to imitate the ischemic events associated with high IOP, as it occurs in glaucoma (Weinreb and Khaw, 2004; Vidal-Sanz et al., 2012). Our goal was to induce ischemic damage directly and locally to the retina and thus to create a progress of inner and outer retinal degeneration from an early stage, right after induction, to a late stage after retinal I/R injury. We wanted to determine the impact of ischemic injury on the entire retina and find out how the damage is developing over the course of time for a better understanding of this degeneration. Therefore, we analyzed six points in time after I/R: 2 h, 6 h, 12 h, 24 h, 3 days, and 7 days. Firstly, we focused on the functionality and structure of the inner and outer retina. We verified a progressive damage regarding both, the function and the appearance. The later the retinae were investigated, the stronger was the degree of ischemic impact on the tissue.

### Impairing of Neuronal Function

Whether impulse transmission is maintained in retinal neurons can be measured by electroretinography. Activity of the photoreceptors (a-wave) and the cells in the INL (b-wave) can be determined with a scotopic ERG. In this context, it should be noted that some studies discuss the relationship between anesthesia and its impact on the ERG outcome (Brown and Green, 1984; Tanskanen et al., 1996; Chaudhary et al., 2003; Nair et al., 2011). The group around Nair et al. (2011), for example, examined ketamine/xylazine, which was used for anesthesia in the current study. They evaluated the effects of this anesthetics on the eye movement and the recorded retinal function. Eye movement could potentially provoke amplitude variations in ERG and thus affect the recordings of functional response. According to Nair et al. (2011) the use of ketamine/xylazine does not completely suppress eye movement. Therefore, in our studies conjuncain is used in addition to anesthetize the eyes topically. This numbs the cornea and counteracts possible eye movements. However, Nair et al. (2011) were also able to show that the greatest a- and b-wave amplitudes in scotopic ERG were detected using ketamine/xylazine. This finding justifies the use of ketamine/xylazine anesthesia as the preferred anesthetic for ERG studies in rats (Nair et al., 2011).

In our study, ERG measurements were performed 3 and 7 days after the ischemic insult and demonstrated a distinct reduction in function at both points in time. During the course of this we noted that the functional restriction increased over time since the decline in amplitudes was stronger after 7 days. In previous studies of our research group where ERG analyses were performed 14 and 21 days after I/R, the size of the a- and b-wave amplitudes was comparable to or even lower than the ones after 7 days (Schmid et al., 2014; Palmhof et al., 2018). This leads to the assumption, that the impairment of function worsens over time. Hence, our data demonstrate that the effect of an ischemic injury spreads out through the retina. Kim et al. (2013) also described a functional disturbance of the retina after ischemic injury. They measured a significant decrease of the a-wave amplitudes first at 21 and 28 days after I/R with a recovery at day 35, which they explain with an early retinal detachment and later reattachment. Nevertheless, the b-wave amplitudes were significantly diminished at day 7 with an ongoing reduction over time until day 35 (Kim et al., 2013). These findings correlate with our data and suggestions. Interestingly, another study from Zhao et al. (2013) revealed a complete loss of the a- and b-wave amplitudes during ischemia, still a decrease at 1 h, and almost recovery of both amplitudes at 48 h after reperfusion (Zhao et al., 2013). However, it should be noted that in comparison to our study the duration of ischemia there was only 17 min. In addition, another model, the so called 4-vessel occlusion model, was used to induce the transient global ischemia and the dark adaption of the animals prior the ERG measurements lasted 70 min instead of 8 h, as in our study. All these factors can influence the outcome. Obviously, the strength and the progress of degeneration vary depending on the type of I/R induction, in particular with regard to the temporal aspect. Considering the period from 2 h to 7 days post I/R, we were able to show for the first time that inducting ischemia by transiently elevating the IOP seems to generate an early and much stronger impairment of function of both, the photoreceptors and the INL cells. In addition, we demonstrated that this damage increases over time until day 7 without any recovery. This leads to the assumption that visual impairment will worsen over time in neurodegenerative retinal diseases associated with ischemic processes. Especially, the longer it remains untreated.

**Table 8C:** Analyses of the rhodopsin area (%) and relative *Rhodopsin* mRNA expression (med) at all points in time.

Rhodopsin						
**Points in time**	**2 h**	**6 h**	**12 h**	**24 h**	**3 days**	**7 days**
**Immunohistology [area fraction in %]**						
Control	100.0 ± 13.4	100.0 ± 21.1	100.0 ± 8.9	100.0 ± 9.9	100.0 ± 6.3	100.0 ± 8.9
Ischemia	126.1 ± 21.3	116.9 ± 35.9	92.4 ± 10.2	81.0 ± 9.2	93.0 ± 5.2	106.3 ± 6.5
*P*-value	0.317	0.703	0.584	0.181	0.407	0.579
**qRT-PCR [fold expression]**						
Relative expression	1.164	0.686	0.621	0.301	0.526	1.108
*P*-value	0.521	0.026	0.007	<0.001	0.008	0.26

*Significant values are marked in red.*

### Progressive Degeneration of RGCs, Retinal Thickness, and the Inner Retinal Layer Including Müller Cell Gliosis

It is well investigated that I/R leads to a significant ganglion cell death (Unoki and LaVail, 1994; Adachi et al., 1996; Weber et al., 1996; Zheng et al., 2007; Wang et al., 2011; Schmid et al., 2014). Also, this cell type reacts particularly sensitive. But the timeline of this degeneration had not been studied in detail yet. Our results show that ischemic processes have such a strong impact, that first signs of an RGC decrease could be detected immunohistologically already 2 h and on mRNA level 12 h after induction. A significant thinning of the GCL was measured as early as 6 h after ischemia. Furthermore, we demonstrated that with advancing examination times, the damage intensified. After 3 days, approximately 75% of the Brn-3a^+^ RGCs were lost and a significant reduction of the whole retinal thickness was observed. These results are consistent with the data from existing publications, which examined a few different points in time post ischemia. 17 min of transient global ischemia led to a reduced number of ßIII-tubulin^+^ RGCs at 6 until 48 h (Zhao et al., 2013). Although the impact of this short ischemia seems to be not strong enough to trigger changes in layer thickness. The authors identified no significant differences in this regard. A study by Sellés-Navarro et al. (1996), where the RGC survival was analyzed at later points in time (5–30 days) after pressure-induced ischemia, indicated RGC loss during the whole study period, starting at day 5. Kim et al. (2013) also performed a pressure-induced retinal ischemia, but in a mouse model. Cell counts in the GCL at five different points in time (3–28 days) revealed a significant decline, with a progressive course, which did not start until day 14. Concerning retinal thickness, a significant thinning of the whole retinal thickness in ischemic eyes was measured beginning at day 21 after injury (Kim et al., 2013). After optic nerve transection and optic nerve crush, RGC death was first observed at day 7 post-lesion and went on until day 15, respectively whereby transection of the optic nerve caused greater damage than the crush (Agudo et al., 2008). Although in these studies the RGC loss was detected at a later point in time post I/R than ours, all this data supports the progressive ischemic-induced damage we were able to prove with our model. Nevertheless, it should be pointed out that in contrast to other studies, we were able to verify the progressive damage of the RGCs and the GCL starting immediately after pressure-induced I/R, indicating the significant extent of this process.

In addition to the GCL, the INL reacts sensitively to ischemic processes. Kim et al. (2013) also described a damage of the INL 3 days after ischemia. Our ERG data indicated that the INL was affected at this point in time, as well. Several studies exhibit, that amacrine as well as bipolar cells are extremely sensitive to ischemic stress (Dijk et al., 2004a,b; Lee et al., 2011; Li et al., 2011). Our group also demonstrated this in previous studies. An ischemic-induced loss of cholinergic amacrine cells was detected after 14 and 21 days (Schmid et al., 2014; Joachim et al., 2017; Palmhof et al., 2018). In addition, significantly fewer glycinergic AII amacrine cells as well as cone bipolar cells were observed 14 days after induction of ischemia (Palmhof et al., 2018). Moreover, Kim et al. (2010) detected a decreased parvalbumin-expressing AII amacrine cell number at later points in time, 4 and 8 weeks after I/R injury. For this reason, it was particularly interesting to examine the course of damage of these cells in the INL at early and late stages post I/R. In this study we could demonstrate that glycinergic AII amacrine cells seem to be more resistant to this damage than cone bipolar cells. While we detected a significant loss of recoverin^+^ cone bipolar cells already at 12 h, the number of parvalbumin^+^ AII amacrine cells decreased significantly later, after 3 days, with a progressive loss over time. Zhao et al. (2013) investigated amacrine and cone bipolar cells in their timeline. They displayed a significantly reduced cell number of calretinin^+^ amacrine cells during the whole study period (6–48 h), but the number of cells between the investigated points in time stayed similar. Concerning recoverin^+^ cone bipolar cells, significantly fewer cells were only noted at 6 and 12 h after ischemic injury (Zhao et al., 2013). The researchers around Dijk et al. (2004b) performed long-term analyses of different sub-types of amacrine and bipolar cells as well. Regarding AII amacrine cells they investigated the expression levels of *Parvalbumin* mRNA via qRT-PCR between 2 h and 4 weeks after I/R induction. Interestingly, they detected a gradual decrease of *Parvalbumin* transcript levels with progressing reperfusion time starting already at 6 h (Dijk et al., 2004b). In case of bipolar cells they focused on rod bipolar cells, which they labeled with PKCα. This sub-type stayed unaffected at all analyzed points in time (2 h–7 days) (Dijk et al., 2004a). A previous study by our group equally revealed no signs of rod bipolar cell degeneration at a late point in time, 21 days after I/R (Schmid et al., 2014). This corresponds to our current results, as the number of PKCα^+^ bipolar cells remained unaltered in the timeframe of our evaluation. These comparisons reveal that the different sub-types of the INL cells, namely of amacrine and bipolar cells, react differently to an ischemic injury. Regarding all underlying findings it can be concluded that bipolar cells are generally more resistant to I/R than amacrine cells. However, relating to the various sub-types we were able to show for the first time, as far as we are aware, that cone bipolar cells are more susceptible against ischemic injury than glycinergic AII amacrine cells. Moreover, we were also able to detect a progressive degeneration of cone bipolar cells over time, for the first time.

Besides retinal neurons, glial cells, like macroglia, are resident in the retina. These include two basic cell types, astrocytes and Müller glia (de Hoz et al., 2016). Pathological conditions such as ischemic damage, neurodegeneration/-inflammation, or trauma lead to an activation of these cells. This process which is associated with an up-regulation of the intermediate filament GFAP, is known as gliosis (Ramirez et al., 2001; Peng et al., 2014). It is reported that transient retinal ischemia leads to Müller cell gliosis (Hirrlinger et al., 2010; Kim et al., 2013). Kim et al. (2013) analyzed 5 points in time (3, 7, 14, 21, and 28 days) via immunohistochemistry and displayed progressive gliosis in retinae that underwent pressure-induced ischemia. Another study by Mages et al. (2019) determined an GFAP up-regulation in Müller cells at 3 days post I/R, with a constantly high level of protein as well as mRNA up to 14 days. We also noted Müller cell gliosis throughout the study. We detected an increased GFAP expression on protein level starting already at 2 h and on mRNA level at 12 h after ischemia induction. Thus, our evaluations correspond with previous published findings. Moreover, we demonstrated that Müller glia are activated at a very early stage of ischemic injury.

### Long-Term Photoreceptor Changes

As mentioned before, most of the analyses regarding the ischemic impact on the retina were performed on the inner retinal layers, due to the assumption that photoreceptors are more tolerant against an ischemia insult. However, previously mentioned data indicate that ischemic damage spreads in the long-term across the whole retina. In addition, analyses of eyes from patients with diabetic retinopathy or of retinae from a rhesus monkey with type 2 diabetes suggest a susceptibility of the photoreceptors. Using adaptive optics scanning laser ophthalmoscopy, photoreceptor abnormalities were found in eyes of diabetic retinopathy patients (Nesper et al., 2017). Regarding the diabetic hypertensive monkey retina, histopathological analyses revealed a severe decline in number of photoreceptor inner and outer segments (Johnson et al., 2005). Therefore, we analyzed both, rod and cone photoreceptors. With regards to the morphology, photoreceptor cones appear to be more sensitive against the ischemic insult than rods. We verified a progressive reduction of opsin^+^ and cone arrestin^+^ cone photoreceptor cells starting at 12 and 6 h post ischemia, respectively. In contrast, no changes in rhodopsin^+^ staining area were noted at all investigated points in time. Indeed, a disorganized structure of rod photoreceptors was observed with progressing examination point in time indicating a structural ischemic damage of rods. Unoki and LaVail (1994) observed shorter and more disorganized photoreceptor outer segments 7 and 14 days after pressure-induced ischemia, indicating a sublethal impairment of photoreceptor cells. The impact of ischemic injury on photoreceptor cones and rods, specifically regarding a temporal degeneration, have hardly been studied so far. A study by Zhao et al. described no alterations in rhodopsin immunoreactivity at 6–48 h after ischemia (Zhao et al., 2013). Our immunohistological results of photoreceptor rods analyses correspond with these findings. However, we measured a significant down-regulation of *Rhodopsin* mRNA expression after 6, 12, and 24 h, and 3 days, which returned to control levels at day 7 after ischemia. Regarding photoreceptor cones, there are so far no investigations in terms of the temporal ischemic influence, to our knowledge. For the first time, we analyzed both photoreceptor cell types, rods and cones, at these early points in time, namely from 2 h on and were able to describe the sensitivity of photoreceptor cones against I/R over time with a progressive course. However, further examinations of this retinal cell type need to be performed, especially as the stimulus processing begins here. In this regard, photopic ERG recordings should be performed in further studies, to investigate cone photoreceptors in more detail.

Since we were able to show a strong ischemia induced damage overall, the association of ischemic extent with anesthesia, especially ketamine, should be referred to, at this point. This subject is part of discussion in some studies, which reported a potential tissue protection by ketamine due to its anti-inflammatory properties (Tsukahara et al., 1992; Frassdorf et al., 2009; Eroglu, 2014). In this regard, myocardial and intestinal injury as well as ischemic injury in the rabbit retina was investigated. Several studies showed that ketamine itself could mitigate ischemic injury and diminish ischemic-induced tissue damage of the different organs (Tsukahara et al., 1992; Frassdorf et al., 2009; Eroglu, 2014). However, the protective effect of ketamine was investigated as a pretreatment therapy. The group of Tsukahara et al. (1992) applied ketamine in the rabbit retina intravitreally 1 h before ischemia induction. In contrast to this, we used ketamine for anesthesia in a cocktail with xylazine and vetranquil via intraperitoneal injection. As we demonstrated such massive retinal injury, it can be assumed that the used dose and application type had no protective effects on the ischemic-induced damage in our study. Moreover, ketamine/xylazine is a common anesthesia for the induction of I/R in rodents (Unoki and LaVail, 1994; Produit-Zengaffinen et al., 2009; Abcouwer et al., 2010; David et al., 2011; Kara et al., 2014; Varga et al., 2017; Lin et al., 2019).

## Conclusion

According to our knowledge, this is the first study to characterize temporal I/R-induced damage throughout the entire retina from the GCL to PRL. Therefore, several cell types of the GCL, INL, and PRL were investigated at six points in time after retinal ischemia. The results demonstrate that ischemic injury, especially pressure-induced I/R, is so intense that it leads to a total retinal deterioration including functional impairment. This retinal ischemic damage appears at a very early point in time, practically right after I/R induction, increases, and spreads with time. In addition to the RGCs, we demonstrated Müller cell gliosis and, for the first time, that cone bipolar and cone photoreceptor cells also seem to be particularly sensitive to I/R, as a progressive cell loss was observed, starting at very early stages. Thus, we suppose that the cone pathway is affected more strongly by ischemic injury. These novel findings should contribute to better understanding of the temporal course of ischemic processes and to the development of new therapeutic approaches. Therefore, the treatment should start as early as possible and include photoreceptors.

## Ethics Statement

This study was approved by the animal care committee of North Rhine-Westphalia (Germany), all experiments were carried out in accordance with the Association for Research in Vision and Ophthalmology (ARVO) statement for the use of animals in ophthalmic and vision research.

## Author Contributions

MP performed experiments, analyzed data, and wrote the manuscript. VF, PR, EK, and JD performed experiments and analyzed the data. NB and GS performed the experiments. HBD revised the manuscript. SCJ designed the study and revised the manuscript. All authors read and approved the final manuscript.

## Conflict of Interest Statement

The authors declare that the research was conducted in the absence of any commercial or financial relationships that could be construed as a potential conflict of interest.

## Abbreviations

AMD: age-related macular degeneration
ERG: electroretinogram
GCL: ganglion cell layer
H&E: hematoxylin & eosin
I/R: ischemia/reperfusion
INL: inner nuclear layer
IOP: intraocular pressure
IPL: inner plexiform layer
ONL: outer nuclear layer
OPL: outer plexiform layer
PRL: photoreceptor layer
qRT-PCR: quantitative real-time PCR
RGCs: retinal ganglion cells
SEM: standard error mean
